# Dimensionality Reduction of Human Gait for Prosthetic Control

**DOI:** 10.3389/fbioe.2021.724626

**Published:** 2021-10-14

**Authors:** David Boe, Alexandra A. Portnova-Fahreeva, Abhishek Sharma, Vijeth Rai, Astrini Sie, Pornthep Preechayasomboon, Eric Rombokas

**Affiliations:** ^1^ Department of Mechanical Engineering, University of Washington, Seattle, WA, United States; ^2^ Department of Electrical Engineering, University of Washington, Seattle, WA, United Staes

**Keywords:** machine learning, kinematic, principal compenent analysis, autoencoder, gait, prosthesis, dimensionality, nonlinear

## Abstract

We seek to use dimensionality reduction to simplify the difficult task of controlling a lower limb prosthesis. Though many techniques for dimensionality reduction have been described, it is not clear which is the most appropriate for human gait data. In this study, we first compare how Principal Component Analysis (PCA) and an autoencoder on poses (Pose-AE) transform human kinematics data during flat ground and stair walking. Second, we compare the performance of PCA, Pose-AE and a new autoencoder trained on full human movement trajectories (Move-AE) in order to capture the time varying properties of gait. We compare these methods for both movement classification and identifying the individual. These are key capabilities for identifying useful data representations for prosthetic control. We first find that Pose-AE outperforms PCA on dimensionality reduction by achieving a higher Variance Accounted For (VAF) across flat ground walking data, stairs data, and undirected natural movements. We then find in our second task that Move-AE significantly outperforms both PCA and Pose-AE on movement classification and individual identification tasks. This suggests the autoencoder is more suitable than PCA for dimensionality reduction of human gait, and can be used to encode useful representations of entire movements to facilitate prosthetic control tasks.

## 1 Introduction

Models of human gait are the foundation upon which lower limb prosthesis controllers are built. Because gait is highly complex and multidimensional, these models take advantage of simplifying assumptions to narrow the problem space. Early above-knee prostheses relied upon events during stance and swing phases to trigger locking and unlocking of a mechanical knee, such as the knee hyperextension moment at toe-off Mauch (1968). As prosthetic technology has advanced, so have the underlying models. Variable damping knees use on-board sensors to detect speed and phase, adjusting knee and ankle joint control parameters to mimic human gait Highsmith et al. (2010). Today, powered prostheses that generate work during gait are gaining in popularity in research circles Azocar et al. (2020). However, the challenge of controlling prostheses has been recently brought again to attention Iandolo et al. (2019) Tucker et al. (2015), and only highlighted by the untapped potential of powered devices to restore mobility. We assert that generating useful representations of human movement is necessary to unlock the potential of such devices.

Gait models can be used to generate reference trajectories of kinematics or torque, or inform a set of control parameters for powered prostheses. Generating safe and reliable trajectories and parameters, given the complexity of human gait, poses a challenge. To do so, simplifications are made. At a high level, activities such as level ground walking, stair navigation, and ramp navigation, can each be called an individual “mode” of movement. At a lower level, control is achieved with respect to phases of gait. For instance, Simon et al. (2014) split the gait cycle of each mode into finite states delineated by gait phases, in which each state corresponds to a set of impedance parameters, totaling 140 tunable parameters. However, adding additional modes creates more tuning parameters, which poses an additional challenge. More recent approaches have reduced the number of tuning parameters by creating unified gait models that span across modes. Quintero et al. (2018) has developed a gait model that generates knee and ankle reference trajectories with respect to speed, phase, and incline. This significantly reduces the solution space while maintaining expressiveness of the model output. However, it remains invariable to idiosyncratic gait characteristics, which Quintero et al. (2018) also identifies as the largest source of variability.

Techniques to simplify gait can be used to address these challenges. One such technique is to reduce the dimensionality of gait by learning its “principal components” from real world data. Dimensionality reduction techniques like Principal Component Analysis (PCA) have been used to identify a variety of pathological gaits Slijepcevic et al. (2018) Matsushima et al. (2017) Chen et al. (2020) Deluzio et al. (1997) and detect differences in kinetics with transfemoral amputation Soares et al. (2016). Unlike standard PCA, nonlinear dimensionality reduction techniques like autoencoders are able to fit a nonlinear function to nonlinear data, though it is unclear which technique is suited for gait—which is a highly structured, periodic behavior. We have previously explored how PCA compares to an autoencoder for dimensionality reduction of hand kinematics, as it pertains to priorities for prosthetic control Portnova-Fahreeva et al. (2020). In this study, we will present a similar analysis using lower limb kinematics collected during gait activities. We will also compare performance between dimensionality reduction techniques on tasks relevant for prosthetic control - movement classification and individual identification.

Movement classification is a broad, albeit powerful, way to simplify gait. For many lower limb prostheses, selecting the desired movement class, like flat ground walking and stair walking, is typically performed via user input, such as bouncing on the heel three times Ottobock (2015). Requiring manual input from the user side steps the challenge of selecting the desired movement class using only sensor inputs. Well performing dimensionality reduction techniques may simplify this challenge, enabling a classifier to automate selection of a movement class, and thus minimizing the control burden placed on the user. However, within a single movement class, gait may be highly variable from one individual to another, either due to pathology, amputation, or idiosyncrasy. These variations are assumed to be chosen to optimize over some set of parameters, like stability Herssens et al. (2020) or metabolic cost Summerside et al. (2018) and so are important to preserve during dimensionality reduction.

Gait models and associated prosthesis control algorithms are also designed to be highly reliable. Because small, rare errors in the model or controller can have catastrophic consequences, simpler solutions are favored. Machine learning algorithms are capable of taking on large, high dimensional problems, but are prone to errors on unseen data and suffer from a lack of interpretability. However, significant interest in machine learning methods over the last decade have resulted in the creation of novel algorithms that offer unique potential for modeling gait. We have previously demonstrated the viability of using machine learning to predict joint kinematics for lower limb prosthesis control Rai et al. (2020) Rai and Rombokas (2019). In this study, we use autoencoders, which are a class of self-supervised networks with flexibility to handle virtually any type of data. We will employ two autoencoders, one trained to reconstruct a single pose from gait (Pose-AE), and a recurrent autoencoder trained to reconstruct an entire movement (Move-AE).

In this study, we seek to better understand how gait data can be simplified using dimensionality reduction. In the first part of this study, we compare the dimensionality reduction performance of PCA on poses and an autoencoder on poses taken from lower limb gait data. In the second part, we will compare performance on movement and individual classification tasks of PCA on poses, an autoencoder on poses, and a recurrent autoencoder on movements. We expect that autoencoders will outperform PCA in all cases, as demonstrated on hand kinematics in our prior work Portnova-Fahreeva et al. (2020).

## 2 Materials and Methods

### 2.1 Data Collection

Gait data was collected in a previous study Rai and Rombokas (2019). Participants wore the Xsens Awinda suit (*Xsens Technologies, Enschede, Netherlands*), a wearable motion capture suit consisting of 17 body-worn sensors. Xsens Analyse software processes raw sensor data to provide joint kinematics in a 3D environment. All angles are in a 1 × 3 Euler representation of the joint angle vector (x, y, z) in degrees, calculated using the Euler sequence ZXY using the International Society of Biomechanics standard joint angle coordinate system Wu et al. (2002). Recruitment and human subject protocols were performed in accordance with the University of Washington Institutional Review Board approval and each subject provided informed consent. De-identified data can be made available, via a data use agreement, upon request to the authors.

From this dataset, we are examining 10 participants who performed flat ground walking and 14 participants who performed stair ascent and descent. All participants in all groups were unique. Flat ground data consists of participants walking at a self-selected speed down a long public corridor. Stair data consists of participants repeatedly descending a wide public 13-step staircase, turning around at the landing, and ascending the same staircase, also at a self-selected speed.

We are also using the Virginia Tech Natural Motion Dataset Geissinger and Asbeck (2020a), also collected using an Xsens system. It contains 40 h of natural, unscripted movement from 17 participants, including 13 participants on a college campus and four participants working in a home improvement store. This dataset is representative of movement in daily life, as compared to constrained activities like steady state forward gait.

### 2.2 Data Processing

Xsens features a real-time engine that processes raw sensor data for each frame and algorithmically fits a human body model to estimate anthropomorphic joint and segment data. A post processing engine includes information from the past, present, and future to get an optimal estimate of the position and orientation of each segment. This “HD” processing raises the data quality by extracting more information from larger time windows and modeling for skin artifacts, etc. but also takes significantly longer time. We used HD processed data as training data for all three datasets.

Each dataset was standardized according to the aggregated statistics of all three datasets. Three lower limb joints on each side of the body (hip, knee, ankle) were chosen for analysis. Each joint can be represented in frontal, transverse, and sagittal planes. Frontal and transverse plane motion was dropped for the knees, due to its propensity to reflect sensor noise over meaningful physiological movement. Because the natural motion dataset contains long periods of inactivity, such as sitting at a desk, it was filtered by pelvic velocity such that only moments punctuated by movement of the pelvis would be included. The subjects of each dataset were then allocated into either train or test sets for analysis. For a comparison study of different techniques, achieving high performing, generalizable results are not our primary aim. Rather, we would like to highlight the attributes of how these techniques interact with the data without optimization. For this reason, we only consider the training set throughout the rest of this study (Table 1).

**TABLE 1:** Details of the training dataset used in this study. Additional individuals were held out for a future testing dataset. Sampled duration reflects the combined length of the recordings from which samples were uniformly extracted.

Dataset	n	Male/Female	Age (yrs)	Height (cm)	Samples	Recording Duration
Flat Ground	8	4/4	26.2 ± 2.7	174 ± 10.9	38,196	1 h 38 min
Stair Walking	11	8/3	24.7 ± 3.5	173 ± 11.2	54,755	2 h 29 min
Natural Movements	13	14/3	20–58	179 ± 7.3	34,779	1 h 36 min

### 2.3 Data Analysis

Data analysis was performed in Python 3.7 and the machine learning was implemented using *Tensorflow 2.0* using a single GPU. Visualization of lower limb poses was achieved using an open source humanoid model in *Unity* (*Unity Technologies, San Francisco, CA, United States*).

#### 2.3.1 Principal Component Analysis

PCA was performed for each dataset using the respective covariance matrix. PCA achieves dimensionality reduction by projecting the original data by the space defined by its principal components (PC), each of which are vectors aligned to maximally capture remaining variation in the data. A limited number of principal components often explain the majority of variation in the data, resulting in a lower dimensional space than the original data. This space will be referred to as the latent space.

#### 2.3.2 Pose Autoencoder

The autoencoder is one variant of the encoder-decoder architecture. Notably, encoder-decoder architectures have been used to power breakthroughs in natural language processing Devlin et al. (2019), but have been applied to computer vision Hossain et al. (2019), time series analysis Lim and Zohren (2021), and human movement Pettee et al. (2019). Pettee et al. (2019) used such techniques to produce manifolds of human dancing, from which samples of novel dance choreography may be decoded. Geissinger and Asbeck (2020b) utilized similar principles to infer complete joint information from sparse sensor input on the natural motion dataset considered here.

Critically, the autoencoder contains a bottleneck through which it is forced to learn features of the data. The activations of the bottleneck layer represent the data in the latent space. Nonlinear activation functions in each layer can capture nonlinear relationships in the data, though often (but not always) at the cost of interpretability.

**FIGURE 1:**
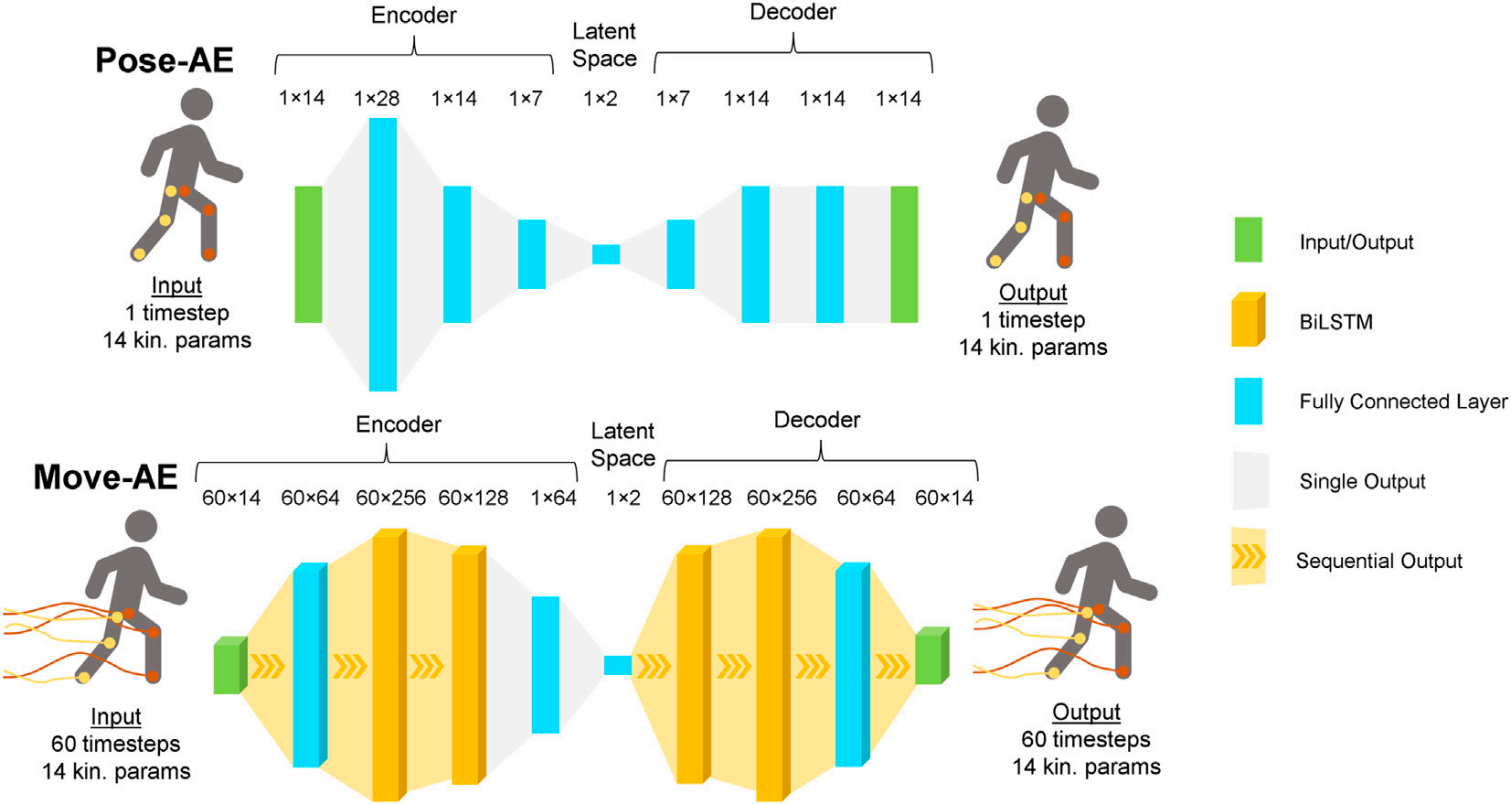
Architecture for the Pose-AE network and Move-AE network. Both networks exhibit the classic autoencoder bottleneck shape. Whereas Pose-AE takes single poses as input, Move-AE makes use of stacked recurrent bidirectional LSTM layers to accept entire movement trajectories as input. Both networks embed each input as a single point in a two dimensional latent space.

As illustrated in Figure 1, inputs to Pose-AE were of size 1 × 14 and consisted of hip, knee, and ankle joint angles, as described in the Data Processing section. For each time series of joint angles, inputs were sampled every 0.166 s. This 1 × 14 vector is then passed through the encoder, after which it can be represented by a 1 × 2 vector in the latent space. The decoder then attempts to reconstruct the original 1 × 14 vector from the 1 × 2 latent vector. The reconstruction error between the decoded 1 × 14 vector and the input 1 × 14 vector backpropagate through the network layers, forcing the network to learn how to best represent the 1 × 14 input vector as a 1 × 2 latent vector. In other words, Pose-AE was trained to reconstruct 14 dimensional “snapshots” of lower limbs from only two dimensions.

Hyperparameter optimization was performed using a single random participant from the flat ground training dataset, with a training and validation split of 50/50. Tuning on a single subject was done as an alternative to k-fold hyperparameter tuning, which becomes combinatorially expensive with three activities. Considering the aim of the study is to compare techniques, not seek maximal performance, the authors decided to err on the side of underfitting, to ensure the most fair comparison across techniques. Hyperparameter choices were found to be insensitive to the chosen subject. We also tested several network widths and depths and found the best results with a three layer block for both the encoder and decoder (Figure 1). Batch normalization was implemented in the encoder to mitigate overfitting. Each configuration was evaluated by its reconstruction loss. Adaptive Moment Estimation was used to optimize learning during training. All Pose-AE models were trained using full batch gradient descent for 8,000 iterations, which was heuristically determined to achieve model convergence before showing evidence of overfitting.

Anecdotally, we discovered very little sensitivity of hyperparameters by subject. T.

#### 2.3.3 Movement Autoencoder

Unlike Pose-AE, Move-AE reduces entire movements. The input to Move-AE was a one second window of all 14 joint kinematics, thus a sequence length of 60 timesteps, given the original 60 Hz recording rate. Though this time series data is higher dimensional, the signal exhibits both autocorrelation and periodicity, making the problem more tractable. Indeed, the fact remains that if one was asked to draw a canonical joint trajectory during flat ground gait of any length, only two pieces of information are required to adequately represent it: cadence and phase. Recurrent layers like the Long Short Term Memory (LSTM) Hochreiter and Schmidhuber (1997) are capable of extracting key information from time series data, and Bidirectional LSTMs Schuster and Paliwal (1997) have been employed here (Figure 1). A point in the latent space now represents one second of movement, rather than a snapshot of a pose. We chose one second of movement as a sufficient length of time to capture the context of a given pose. This is in contrast to the pose autoencoder and PCA, which would be unable to determine if one was walking forward or backward because they cannot learn the temporal dependencies within movements.

Inputs to Move-AE consisted of the same 14 joints and planes as Pose-AE, but now extend to include 1 s of movement at 60 Hz. These inputs are similarly sampled every 0.166 s, regardless of gait phase, meaning there is overlapping data between multiple inputs. The input vector is now shaped 60 × 14 (60 timesteps, 14 joints and planes). As before, the 60 × 14 input vector passes through the encoder, after which it is represented as a 1 × 2 latent vector. The decoder reconstructs the entire 60 × 14 input from this 1 × 2 latent vector. Move-AE learns to represent entire movement trajectories of the lower limbs as a 1 × 2 vector.

Hyperparameter optimization was performed as previously described. The best performing architecture was found to have two bidirectional LSTM (biLSTM) layers for both the encoder and decoder and time distributed fully connected layers before and after. Making a fully connected layer “time distributed” allows it to accept sequential data by passing each timestep through individually. Time distributed fully connected layers of width 64 are used to generate the input sequence to the encoder biLSTM block, and generate the output sequence from the decoder biLSTM block. An intermediate fully connected layer was included after the encoder biLSTM block to facilitate dimensionality reduction to two dimensions in the latent layer. All models were trained for 8,000 iterations as previously described, except we used mini batch gradient descent with a batch size of 32 to decrease training time with larger inputs.

#### 2.3.4 Variance Metrics

As described in our previous work Portnova-Fahreeva et al. (2020), Variance Accounted For (VAF) is a measure of how well a model reconstructs an input from the latent space. A VAF of 100% indicates the reconstructed output is identical to the input. VAF was evaluated for inputs reconstructed by PCA and autoencoder for each dataset. The equation is presented again here for clarity (Equation 1).

$$VAF\left(\%\right)=\left(1-\frac{var\left(y-\hat{y}\right)}{var\left(y\right)}\right)*100$$ (1)

We compare how variance is distributed between each dimension of the latent space. Principal components were ranked by variance explained and normalized to the sum of previous principal components, thus converting the variance of each PC into a ratio of the total variance. The autoencoder was trained using various bottleneck widths. Similarly, the variance of activations in the bottleneck were normalized to the sum of all variances in the bottleneck layer. As per previous results Portnova-Fahreeva et al. (2020), dimensional variance is expected to be more uniformly distributed in the autoencoder, which does not have the constraint of PCA’s orthonormality, but is capable of sharing variance across multiple latent dimensions. We also report the Root Mean Square Error (RMSE) between the original input and the reconstructed input. Note how RMSE differs from VAF, in that it directly measures error in reconstruction, whereas VAF measures what proportion of variance has been captured.

#### 2.3.5 Classification Tasks

We compare the performance of all three dimensionality reduction methods on two classification tasks: movement classification and individual identification. For both tasks, we use a Support Vector Machine to classify within the latent space, performed using the scikit-learn Pedregosa et al. (2011) implementation, in turn based on the formulation presented here Chang and Lin (2011). We use a radial basis function kernel to improve classification accuracy, given the low-dimensional latent space. All parameters were fixed for all tasks and latent spaces.

The movement classifier sought to determine whether a given point in the latent space represented flat ground walking or stairs navigation. The natural movement dataset was excluded due to the presence of both activities within the single dataset. The individual classifier sought to identify the individual from which a given input in the flat ground walking dataset originated. The error was calculated as the number of erroneously classified inputs divided by the total number of inputs. In both cases, the training dataset was used, and training was repeated 10 times for each model to capture a better range of outcomes. The Kolmogorov-Smirnov (KS) test was employed to test if Pose-AE and Move-AE classification errors were drawn from different distributions. The KS test is well suited when the sets under comparison exhibit different variances.

The classification results indicate how separable different movements and individuals are in the latent space. High separability will result in high classification accuracy, indicating that the dimensionality reduction technique has preserved high amount of information about the input. This test also allows a direct comparison to be made across techniques as diverse as PCA, autoencoders, and recurrent time-sensitive autoencoders.

## 3 Results

### 3.1 Dimensionality Reduction

**FIGURE 2:**
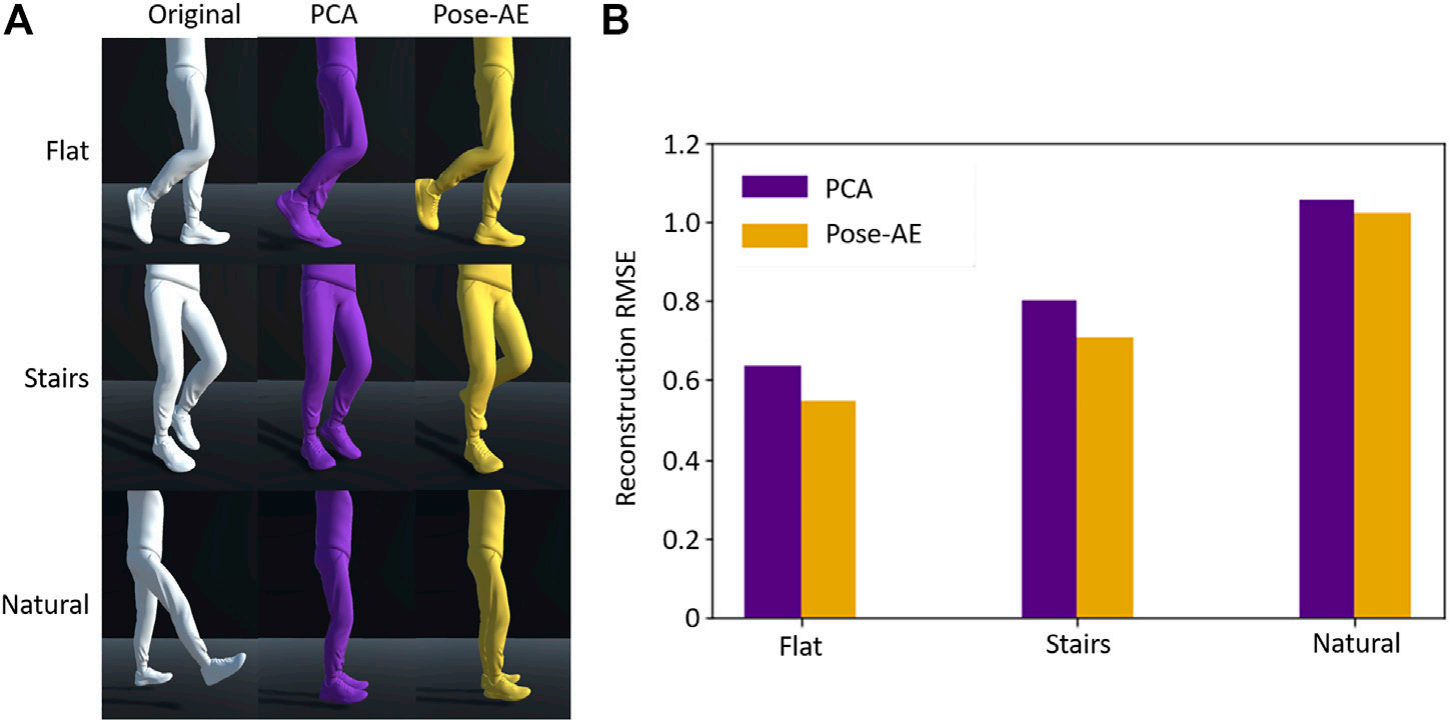
**(A)** For each dataset, a randomly chosen sample was reconstructed using PCA and Pose-AE. Pose-AE produced qualitatively improved poses over PCA, though neither was able to reconstruct poses from natural movements dataset, instead electing to reconstruct a mean standing pose. **(B)** RMSE of joint angles was calculated for each dataset and method. Pose-AE shows a modest improvement over PCA.

Pose-AE exhibited better pose reconstruction than PCA for flat ground and stair walking, but neither Pose-AE nor PCA was able to adequately reconstruct natural poses from a two dimensional latent space (RMSE 0.63 vs 0.55, 0.80 vs 0.71, 1.05 vs 1.02) (Figure 2). For flat ground and stair walking, visual inspection of randomly chosen reconstructed poses by each method illustrate how even small improvements in RMSE may result in qualitatively improved pose reconstruction, especially in regards to sagittal plane. However, both methods perform poorly on the natural movement dataset. An RMSE >1 indicates neither method is an improvement from simply reconstructing the mean pose.

**FIGURE 3:**
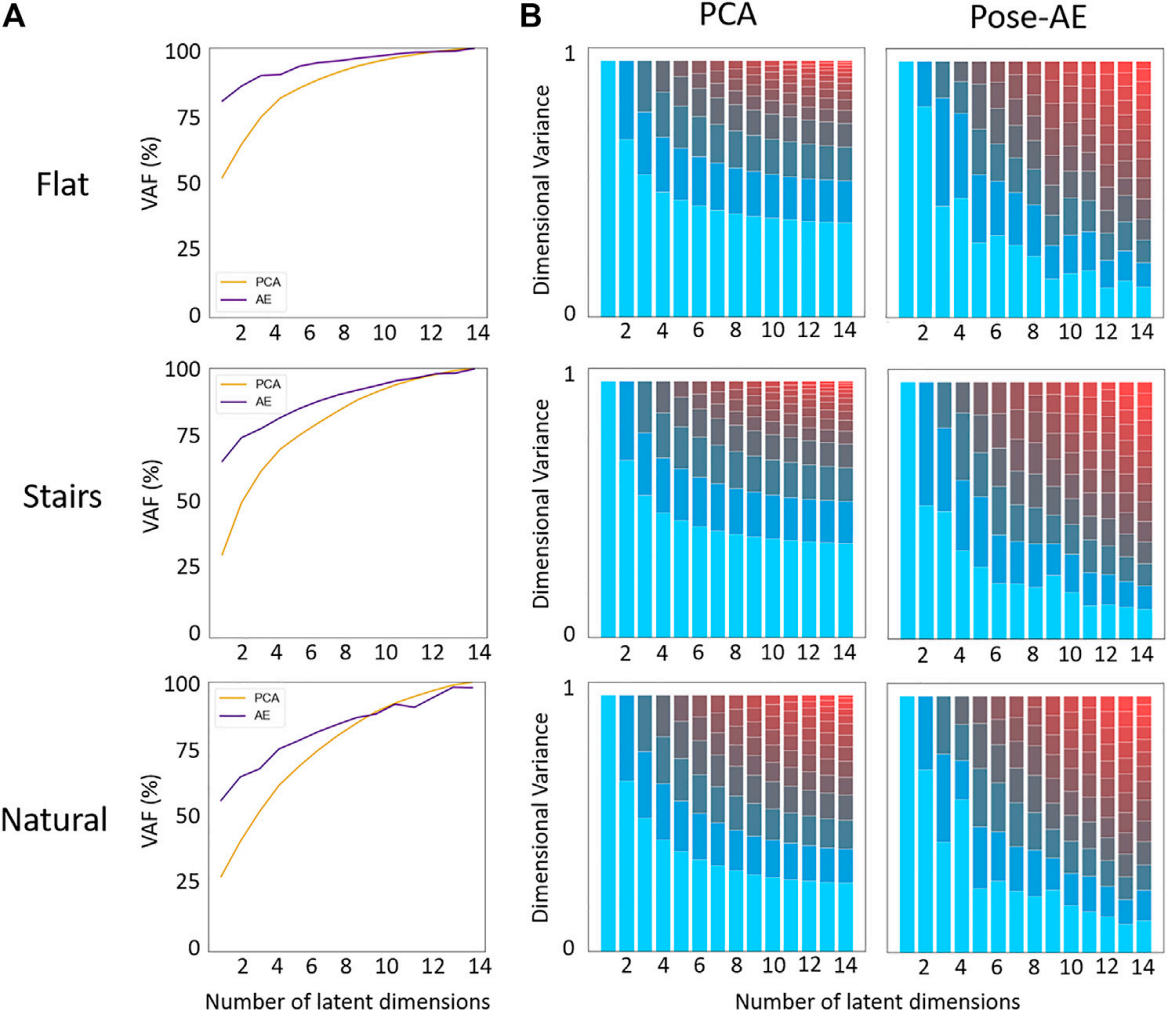
**(A)** Pose-AE captured more variation in the data than PCA for all three datasets. **(B)** Dimensional variance is more evenly distributed with Pose-AE than PCA. The effect is most pronounced with higher dimension latent spaces. The largest dimension by variance has been highlighted blue to facilitate comparison across PCA and Pose-AE.

Similar to previous findings Portnova-Fahreeva et al. (2020), Pose-AE captures greater variance in the data than PCA during dimensionality reduction, especially at low dimensions (Figure 3). Dimensional variance is more evenly distributed with Pose-AE than PCA. Though neither method were suited to reconstruct natural movement poses, Pose-AE retained an evenly distributed dimensional variance - indicating the capability to share dimensional variance across dimensions is inherent to autoencoders, regardless of dataset.

### 3.2 Movement Separability

**FIGURE 4:**
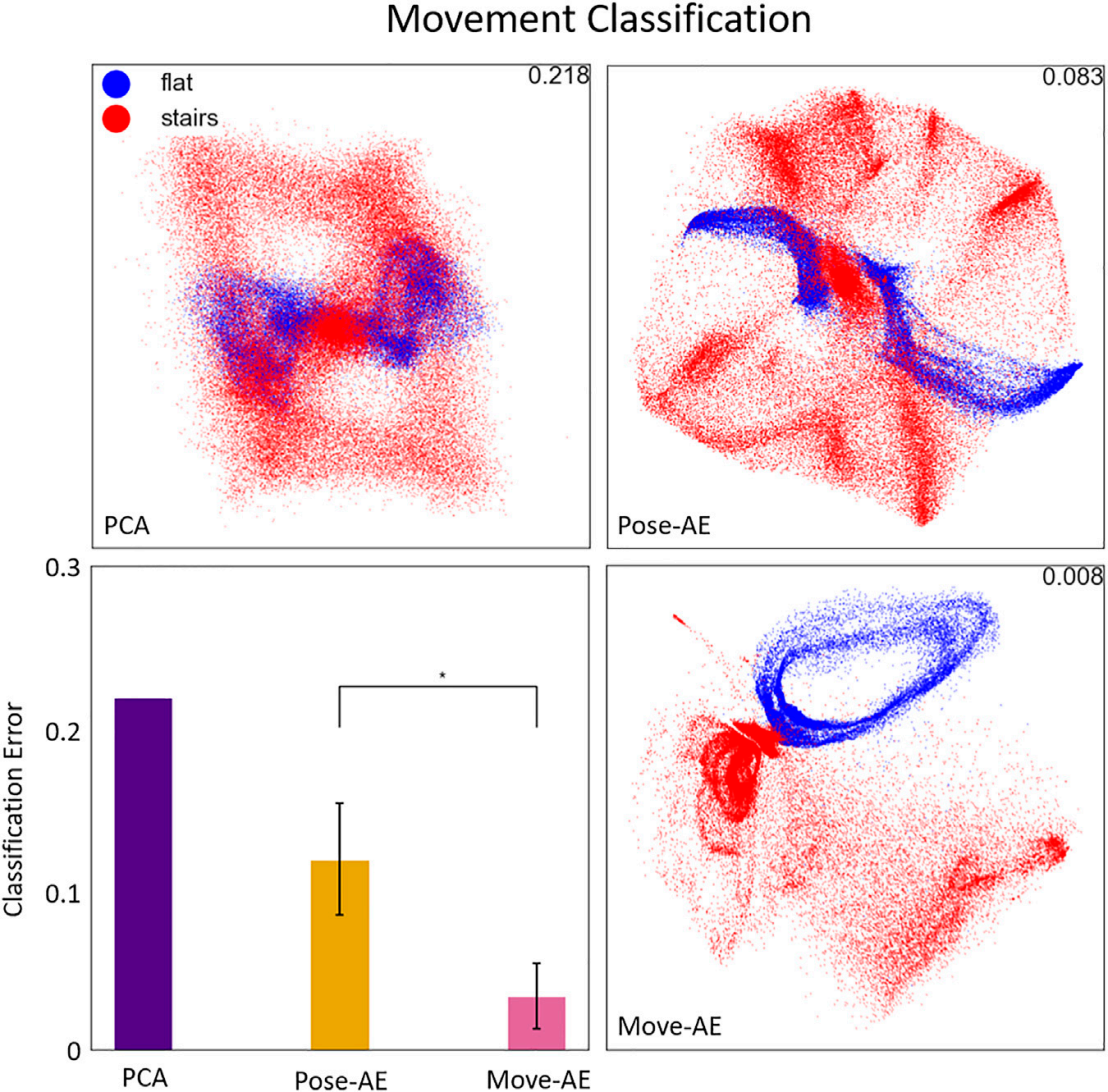
The three panes display the best performing latent space of their respective method. The latent space is a visualization of the activations of the two coding units in the bottleneck, or the first two principal components. The bottom-left pane shows the results of a movement classifier SVM trained on the latent spaces for each method. Error bars denote the varying performance from each of 10 runs for both autoencoders. The Move-AE latent space outperformed Pose-AE and PCA latent spaces on classifying between flat ground and stair walking. Recall that in the Move-AE latent space, each point represents an entire movement. Flat ground walking is embedded in a cyclical structure that is well separated from stair walking.

Both autoencoder models produced latent spaces more suited for movement classification than PCA (error 21.8% PCA; 11.7 ± 3.4% Pose-AE; 3.3 ± 2.0% Move-AE). Move-AE exhibited significantly different movement classification performance than Pose-AE (KS test, *p* < 0.0001). The latent spaces of each method’s best performing model are visualized in Figure 4. Unlike PCA, Pose-AE was sensitive to differences in flat ground walking and stair walking, thus embedding them with little overlap in the latent space. Though Move-AE was trained to compress its inputs by a much larger ratio (420:1 for Move-AE vs 7:1 for Pose-AE and PCA), it was able to embed whole movements in different regions of the latent space without explicit labels. We observed many variations in how the data were embedded in the latent space between each of the 10 runs, especially for Move-AE, hence the increased variability in classification performance.

### 3.3 Individual Identification

**FIGURE 5:**
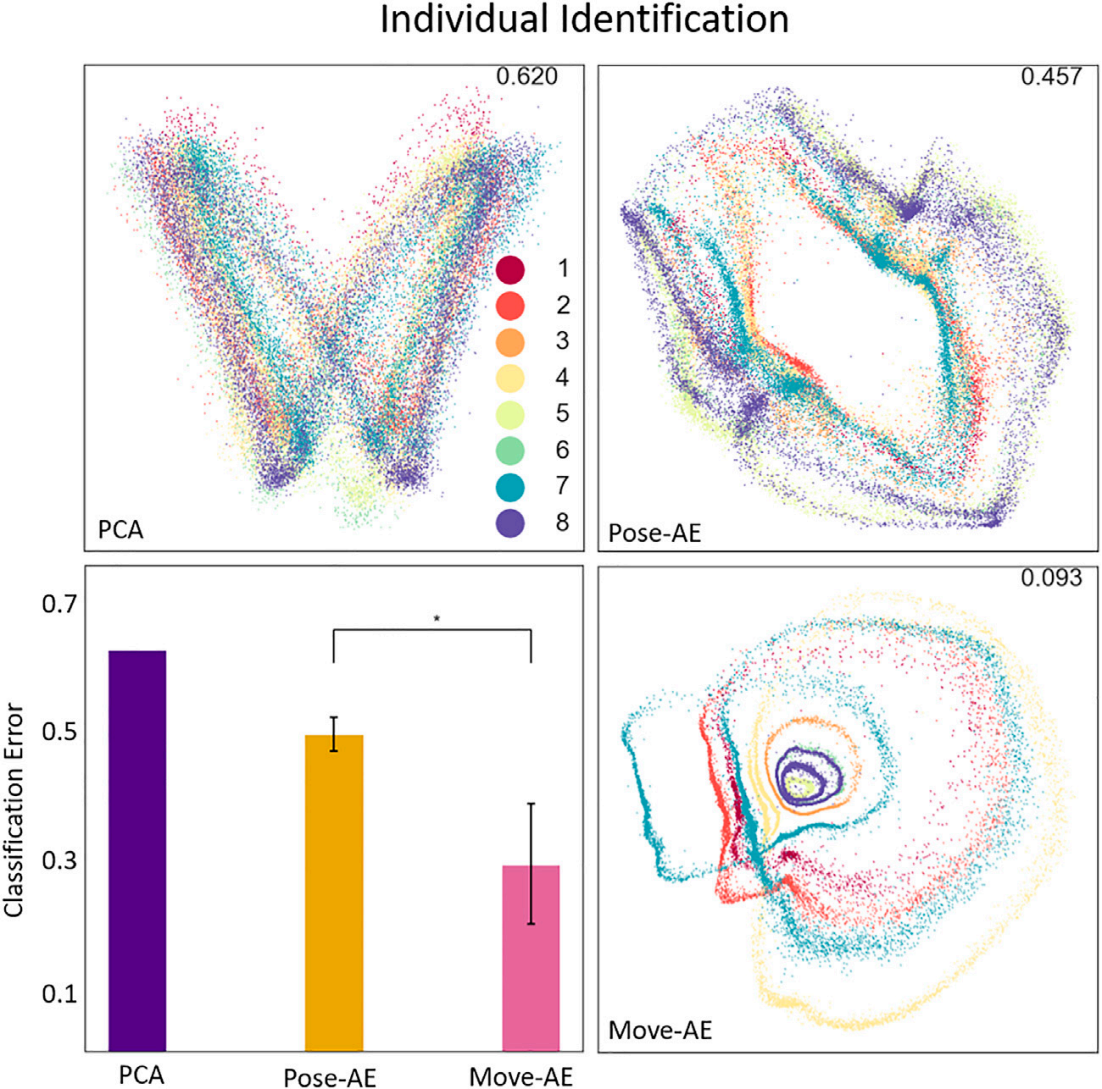
The three panes display the best performing latent space of their respective method. The plotted data is colored by one of eight individual subjects. The classification error for the best performing model is included in the top-right of each plot. The bottom-left pane shows the results of an individual-specific classifier SVM trained on the latent spaces for each method. Note that the Move-AE latent space here is a well-performing outlier, though visual inspection of the other latent spaces confirm the general behavior of separately embedding individuals.

Similar to movement classification, Move-AE outperforms Pose-AE (KS test, *p* < 0.0001), which in turn outperforms PCA on classification of individuals (error 62.0% PCA; 48.9 ± 2.6% Pose-AE; 28.9 ± 9.3% Move-AE). All three methods produce cyclical representations of gait within their latent spaces, but Move-AE also cleanly separates between many individual gaits, again without providing an explicit label (Figure 5).

## 4 Discussion

Understanding the high dimensionality of human gait remains a significant challenge but may yield an equally significant payoff. Creating a useful low dimensional representation of gait may serve to benefit both gait analysis and control of a device. Many techniques for dimensionality reduction exist, though PCA has remained popular for its ease of implementation and interpretability. However, our results indicate that autoencoders are better suited for reducing human movement on both performance measures of reconstruction and Variance Accounted For. Relationships with human gait features, and biological data in general, is generally nonlinear. The nonlinear activation function within neural networks enables them to capture such relationships, whereas PCA cannot. Nonlinear PCA methods like Kernel PCA may be better suited than standard PCA for such tasks Mika et al. (1998).

We employed three datasets of increasing complexity. Flat ground walking is the least complex, in that it only contains cyclic steady-state gait. Stair walking is more complex, as it contains upstairs and downstairs segments, and the transitions in between. Natural movements are most complex, in that they contain both cyclic movement and non-cyclic movements, with a variety of actions being performed. As data becomes more complex, the advantage of autoencoders over PCA is diminished (Figure 2). This may be due to the tradeoff between quantity and variety of movements within each dataset, thus the autoencoder is impoverished of sufficient examples of more complex movements from which to learn. Thus, care should be taken to carefully curate the activities within smaller datasets to achieve good dimensionality reduction. For instance, composition of the training data should be deliberately balanced to match the desired performance on each example. Movements that appear rarely will not affect the gradient sufficiently to achieve adequate reconstruction, whereas movements that appear too often will dominate the gradient at the expense of others.

Incorporating temporal context in the input to Move-AE dramatically enhances its capability to discriminate between movements and individual gait profiles. This is understandable considering how given a full second of gait, or about one full gait cycle, whatever differences that exist between individuals or movements will be present within every input. This capability is not afforded to standard PCA, which can only operate on n-dimensional vectors, rather than mxn-dimensional matrices. Interestingly, producing Move-AE also had some unintended consequences. For instance, while performing the individual identification task, it became apparent that half of subject 7’s flat ground data was persistently embedded separate from all others in the latent space. Upon visual inspection of the data, it was apparent there was a minor sensor calibration or data processing error that was small enough to escape detection until that moment (offending data was removed and all trials repeated without it).

We show that it is possible to reliably classify movements and some individuals using the Move-AE architecture. However, without retraining the network on an equivalent dataset of actual prosthesis users, it is unknown how effective such a strategy may be in practice. Nevertheless, automating the selection of modes, or perhaps gait parameters, reduces the control burden on the users of mode-based prostheses, who must perform unnatural motions with their prosthesis to select the right mode for the terrain. We also show that Move-AE is sensitive to individual gaits. These variations, arising from the dynamic cost landscape of walking, are important to preserve. This also portends that such a network will be able to capture the dynamics of pathological or compensatory gait, embedding them within discrete latent structures. However, organization of individuals in the latent space is not necessarily meaningful - sampling halfway between two individuals in the latent space may not produce a pose or movement that is halfway between them in euclidean space.

It should be noted that the aim of the classification task presented here is not intended to maximize classification performance of movements. There are other, better suited methods to achieve high classification accuracy, when labelled data is available. Rather, we designed the task to compare the relative capacity of each dimensionality reduction method to preserve valuable information like movement class or individual gait. Future work is needed to determine how such methods perform on unseen movements and individuals. Autoencoders in particular tend to “de-noise” unseen data such that it better resembles the data on which they were trained Vincent et al. (2008).

Although dimensionality reduction techniques as described here are powerful tools to simplify and analyze gait data, they are not sufficient to achieve prosthetic control alone. An autoencoder on movement data only serves to make sensor data more palatable—it does not provide its own inference about the data. For instance, the Move-AE architecture self-supervises to embed movements in the latent space, but does not classify without an additional classifier like an SVM. Our results show that such learned embeddings can automatically separate movements in the absence of any goal but reconstruction. Future work is needed to move the application of these tools from offline analysis to online integration with a controller. In a practical, online scenario, it is still unknown the quantity, variety, and richness of data required from an individual walker to train a personalized Move-AE architecture to satisfaction towards a given task, like movement classification. Indeed, this discussion focuses on facilitation of “high-level” prosthetic control, or mode selection, rather than “low-level” control over moment to moment commands to the actuator which remains critically important. Furthermore, our results may still be replicable using raw inertial measurement unit data from a few strategically placed sensors on the lower limbs, rather than the full wearable motion capture system used here, in line with the work by Geissinger and Asbeck (2020b).

We have demonstrated that autoencoders can generate structured, interpretable latent spaces. This class of self-supervised networks are able to learn without hand-crafted labels, making them suitable to tackle complex problems like human movement. For instance, latent representations of gait form cyclic structures organized by phase, without human intervention to segment the gait data. Though not presented here, the authors found that distance to the center of the cyclic structure corresponds directly with cadence—faster cadences form tighter rings, slower cadences form larger ones. Contrary to the popular notion that neural networks are a black box, autoencoders can produce structured latent spaces, and thus could be incorporated into prosthetic controllers, either to simplify incoming sensor data, or to generate movement commands via sampling in the latent space.

Interpretation of latent spaces is fast becoming an important topic of research as neural networks become more prevalent. It should be noted that sampling from these latent spaces may enable generation of individual-specific synthetic gait cycles. For instance, sampling points from a Gaussian distribution centered on the region where mid-swing is embedded in the latent space may produce multiple variations of a mid-swing trajectory in the decoder, as learned from training data. Further research is needed to determine how best to create a latent space that lends itself to sampling - as stated previously, distances within an autoencoder’s latent space are not necessarily meaningful. Sampling meaningful movements from the latent space is a non-trivial problem, in part due to the difficulty in describing the latent space’s geometry, or manifold. Application of adversarial or variational autoencoders, which enforce additional distributional constraints on the latent space, may be key to building sample-suitable latent spaces.

Useful representations of gait are a necessary ingredient for leveraging the power of machine learning for prosthetic control. This study shows how autoencoders may create such a representation purely from data, and crucially, are capable of handling temporal data.

## Data Availability

The raw data supporting the conclusions of this article will be made available by the authors, without undue reservation.
